# The choroidal macrophage polarization significantly influences myopia development in murine models

**DOI:** 10.1016/j.isci.2026.115764

**Published:** 2026-04-17

**Authors:** Jing Hou, Shin-ichi Ikeda, Yajing Yang, Tomokazu Fukuchi, Chiaki Ikeda, Satoshi Imanishi, Ziyan Ma, Junhan Chen, Kiwako Mori, Hidemasa Torii, Hideki Fujii, Kazuno Negishi, Kazuo Tsubota, Toshihide Kurihara

**Affiliations:** 1 Laboratory of Photobiology, Keio University School of Medicine, 35 Shinanomachi, Shinjuku-ku, Tokyo 160-8582, Japan; 2 Tsubota Laboratory, Inc., 35 Shinanomachi Shinjuku-ku, Tokyo 160-8582, Japan; 3 Department of Ophthalmology, Keio University School of Medicine, 35 Shinanomachi, Shinjuku-ku, Tokyo 160-8582, Japan; 4 Department of Biology, Keio University School of Medicine, 4-1-1 Hiyoshi, Kohoku-ku, Yokohama 223-8521, Kanagawa, Japan

**Keywords:** Immunology, Cell biology

## Abstract

While choroidal macrophages regulate homeostasis in macular degeneration, their role in myopia is unclear. In this study, depleting choroidal macrophages via clodronate liposome injections induced myopia. To further dissect their function, introducing polarized classically activated (M1) or alternatively activated (M2) macrophages into a myopia model revealed that both the presence and polarization of choroidal macrophages affect myopia progression. Further exploration in normal mice showed that choroidal M1 macrophages polarization triggered choroidal thinning and promoted myopia progression, whereas M2 macrophages polarization enhanced choroidal thickening and suppressed myopia development. These opposite effects of M1 and M2 macrophages on choroidal thickness and myopia progression appeared to be related to their differential regulation of inflammatory responses and oxidative stress. Taken together, these results support a role for choroidal macrophages and their polarization states in myopia onset and progression, suggesting potential therapeutic strategies for controlling the development and progression of myopia.

## Highlights

• Choroidal macrophage depletion triggers choroidal thinning and myopia onset
• M1 and M2 macrophages exert opposite effects on myopia progression
• Macrophage polarization regulates choroidal inflammation and oxidative stress
• Modulating macrophage phenotypes offers a new therapeutic strategy for myopia

## Graphical abstract

**Figure:**
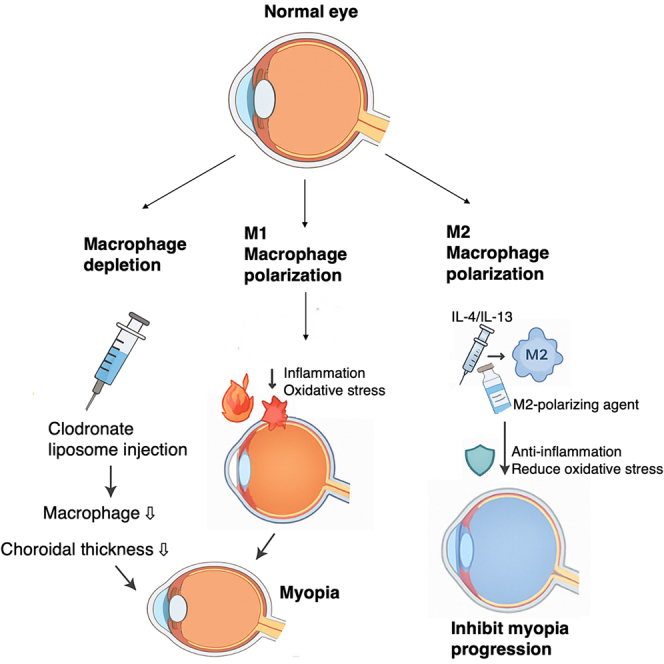

## Introduction

Myopia, commonly referred to as “short-sightedness,” is the most prevalent vision-related disease worldwide, which is caused by refractive error depending on the elongation of the eyeball along the visual axis.^1^^,^^2^^,^^3^^,^^4^ Existing research has shown that myopia is affected by a variety of factors, including light environmental impacts, dietary factors, and genetics; however, the pathogenesis of myopia remains unclear.^5^^,^^6^^,^^7^^,^^8^ Numerous studies have suggested that the inflammatory response plays a role in the development of myopia. Specifically, some studies have indicated that increased inflammatory reaction in the eye accelerates the development of myopia, whereas anti-inflammatory reactions inhibit it.^9^^,^^10^^,^^11^^,^^12^ Notably, macrophages play indispensable roles in the initiation, maintenance, and resolution of inflammation.^13^

As key constituents of the innate immune system, macrophages are present in virtually all mammalian tissues and display remarkable functional diversity. Beyond their immunological roles, they also contribute critically to tissue development and the preservation of homeostasis..^14^^,^^15^ In the ocular system, a healthy cornea contains a population of macrophages that maintain corneal lymphatic homeostasis and promote lymphangiogenesis under inflammatory conditions.^16^ Confined to the inner retina under homeostatic conditions, microglia, the resident macrophages of the neural retina, play a central role in preserving retinal integrity.^17^^,^^18^^,^^19^ Studies have shown that the choroid harbors a dense network of tissue-resident macrophages.^20^ These resident dendritic macrophages are closely aligned along the vessel walls in choroidal arteries and arterioles. Previous work has demonstrated that in the choroidal terminal choriocapillaris, macrophages are not tightly packed on the vessel wall but are widely distributed in the connective tissue between blood vessels.^21^ These findings provide a basis for investigating the relationship between choroidal vessels and macrophages. Subsequently, researchers revealed that the ablation of a considerable majority of choroidal macrophages was associated with broad and gradual atrophy of the choroidal vasculature and thinning of the choroid.^22^ This finding indicates that choroidal macrophages play an important role in the maintenance of the choroidal vasculature. Existing research on myopia has established that the stability of the choroidal vasculature is essential for maintaining choroidal thickness, and choroidal thickness variation is a structural characteristic of the development of myopia.^23^^,^^24^^,^^25^^,^^26^ Considering these findings, we hypothesized that choroidal macrophages play a crucial role in the progression of myopia.

Macrophages exhibit remarkable phenotypic plasticity and can adopt distinct functional states in response to microenvironmental cues. Among the various classification frameworks, the M1/M2 polarization model remains widely used to describe pro- and anti-inflammatory macrophage subsets..^27^^,^^28^ Classically activated (M1) macrophages are typically induced by microbial products and pro-inflammatory signals, and are characterized by the production of inflammatory cytokines such as tumor necrosis factor-α (TNF-α) and interleukin-6 (IL-6), along with elevated levels of reactive oxygen species (ROS). In contrast, alternatively activated (M2) macrophages arise in response to anti-inflammatory stimuli and contribute to the resolution of inflammation by suppressing ROS production and promoting tissue repair.^29^^,^^30^^,^^31^ Studies have shown that inflammatory diseases increase the risk of myopia and that the administration of pro-inflammatory cytokines to the eye contributes to the progression of myopia.^12^^,^^32^ These findings underscore the importance of controlling inflammatory response in inhibiting the development of myopia. Moreover, it has been suggested that the existence of several supplements that convert M1 into M2 macrophages.^33^^,^^34^^,^^35^ Therefore, we hypothesized that different subsets of macrophages in the choroid have diametrically different effects on the development of myopia depending on their characteristics and polarization direction.

In this study, we examined the role of choroidal macrophages in myopia development. We found that macrophage depletion promoted axial elongation and that polarization direction influenced myopia progression. These findings suggest that immune-related mechanisms contribute to myopia and that macrophage phenotype modulation may be relevant to myopia control.

## Results

### Sustained intraperitoneal injection of clodronate liposome depletes choroidal macrophages and triggers myopia

**Figure 1:**
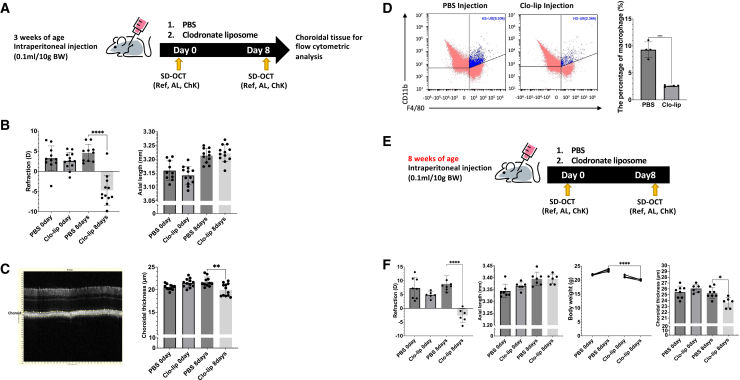
Myopia is accompanied by the sustained depletion of choroidal-resident macrophages in mice at 3 and 8 weeks of age (A) Experimental design (3 weeks). Wild-type C57BL/6J1 mice (*n* = 6 mice per group) were intraperitoneally injected (0.1 mL/10 g) with clodronate liposomes (clo-lip) or control liposomes every two days (four injections) over eight days. Refraction (Ref), axial length (AL), and choroidal thickness (ChK) were measured at baseline and after the final injection using infrared photorefraction and SD-OCT. (B) The clo-lip group exhibited significant myopia shift (*p* < 0.0001) and axial length elongation. Individual data points represent single eyes; Bars represent mean +/− standard deviations. (C) B-scan obtained by SD-OCT was used for choroidal thickness measurement; the yellow delineated region was manually traced in ImageJ to represent choroidal thickness. Clodronate liposome treatment significantly reduced choroidal thickness at Day 8 compared with PBS controls (*p* < 0.01). (D) Flow cytometric analysis revealed a significant reduction in CD11b^+^F4/80^+^ macrophages (H3-UR region) in the clo-lip group (*p* < 0.0001). Representative flow cytometry plots from three independent experiments are shown. (E) Experimental design (8 weeks). Wild-type C57BL/6J1 mice (*n* = 4 per group) received the same injection regimen as described in (a). (F) In 8-week-old mice, 8 days of clo-lip treatment also led to significant myopia shift (*p* < 0.0001), greater axial elongation, and reduced choroidal thickness (*p* < 0.05) compared with controls. Data are mean ± SD. Differences were evaluated using two-way ANOVA + Tukey post hoc. *∗p < 0.05*, *∗∗p < 0.01*, *∗∗∗p < 0.001*, and *∗∗∗∗p < 0.0001.*

To investigate the effect of resident macrophage depletion on refractive status in a mouse model, 3-week-old C57BL/6J mice were subjected to intraperitoneal injection with clodronate liposome (clo-lip) or phosphate-buffered saline (PBS) liposomes as a control treatment (Figure 1A). Detailed reagent information is provided in the Supplemental Table. Baseline refractive error, axial length, and choroidal thickness were comparable between PBS and clo-lip-treated mice prior to treatment (Figures 1B and 1C). Therefore, changes in these parameters from baseline were calculated and used to assess myopia progression throughout the study, unless otherwise indicated. The mice treated with clo-lip exhibited a higher myopic shift in refractive status (−7.30 ± 3.61 D vs. +1.17 ± 4.40 D, *p* < 0.01), greater axial length elongation (0.08 ± 0.02 mm vs. 0.05 ± 0.02 mm, *p* < 0.01), and greater choroidal thinning (−1.56 ± 0.89 μm vs. 1.09 ± 0.51 μm, *p* < 0.0001) than the PBS liposome-treated mice (Figure S1A). Flow cytometry results also showed that choroidal macrophages were significantly depleted after the clo-lip injection (9.30% ± 0.018 vs. 2.58% ± 0.002, *p* < 0.0001) (Figure 1D). Previous studies have shown that choroidal thickness in mice does not increase and tends to remain stable at approximately 8 weeks of age in mice.^36^ To investigate whether the depletion of choroidal macrophages still induces myopia in mice with stable choroidal thickness, 8-week-old adult mice were injected with clo-lip or PBS liposomes using the above-mentioned procedure (Figure 1E). In adult mice, clo-lip treatment induced a significant myopic shift and choroidal thinning compared with PBS controls. However, axial elongation was modest and was significantly influenced by changes in body weight following clo-lip administration. After normalizing axial length changes to body weight, the direction of myopia-related ocular changes remained consistent with those observed in juvenile mice (Figures 1F and S1B). These results suggested that systemic macrophage depletion can induce myopia in mice, regardless of whether they have stable choroidal thickness or not.

### Suprachoroidal space injection of clo-lip specifically depleted choroidal macrophages and induced a pronounced myopia

**Figure 2:**
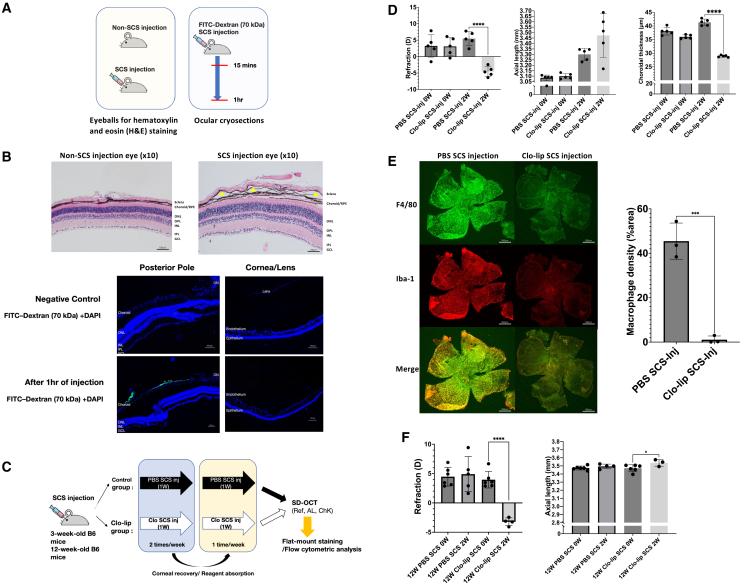
Suprachoroidal injection of clodronate liposomes induced myopia by depleting choroidal macrophages (A) Schematic illustration of subconjunctival space (SCS) injection and tracer diffusion assay. Eyes receiving SCS injection or non-SCS injection were collected for hematoxylin and eosin (H&E) staining. To assess ocular tissue permeability following SCS injection, FITC-dextran (70 kDa) was administered via SCS injection, and choroidal and retinal tissues were harvested at 15 min and 1 h for flat-mount analysis. (B) Representative images from three independent experiments are shown. Histological comparison of eyes that received SCS injections versus uninjected controls revealed an enlarged suprachoroidal cavity (yellow arrowheads) without detectable retinal damage. Representative images are shown (10× magnification). Scale bars, 100 μm. Cryosections demonstrate the preferential accumulation of FITC-dextran in the choroid, did not diffuse into retina, cornea, anterior segment, and even aqueous humor after 1h of injection, indicating selective delivery to the choroidal compartment. Scale bars, 100μm. (C) *Experimental design.* Three-week-old wild-type C57BL/6J1 mice (*n* = 5 per group) received PBS or clo-lip via SCS injection twice per week for two weeks. Ref, AL, and ChK were measured by SD-OCT, followed by flat-mount staining and flow cytometric analysis. (D) Compared with PBS SCS injection, clodronate liposome SCS injection induced a significant myopic shift (*p* < 0.0001) and choroidal thinning (*p* < 0.0001) after 2 weeks. Individual data points represent single eyes; Data are presented as mean ± SD. (E) Representative images from three independent experiments are shown. Immunofluorescence of sclerochoroidal whole mounts stained with F4/80 (green) and Iba-1 (red) demonstrates reduced macrophage density in clo-lip-treated eyes. Scale bars, 500 μm. Panels show representative images with 2× insets. Quantification confirms significantly reduced macrophage density in clo-lip-treated mice (*p* < 0.001). Scale bars, 500μm. (F) Effects of SCS macrophage depletion in 12-week-old mice. clodronate liposome SCS injection induced a significant myopic shift (*p* < 0.0001) and axial elongation (*p* < 0.05), indicating reduced susceptibility to macrophage depletion-induced myopia in adulthood. Data are presented as mean ± SEM. *p* values were determined using unpaired two-tailed t-tests/two-way ANOVA + Tukey post hoc. ∗*p* < 0.05, ∗∗*p* < 0.01, ∗∗∗*p* < 0.001, and ∗∗∗∗*p* < 0.0001.

Intraperitoneal injection of clo-lip not only depleted macrophages in the choroid but also potentially affected macrophages in other tissues. We injected clo-lip into the suprachoroidal space (SCS) to evaluate the effect of choroidal macrophages on the progression of myopia. Figure 2A illustrates the experimental workflow for evaluating the tissue distribution and local effects of SCS injection, including histological assessment and FITC-dextran tracing. The presence of multiple cavities in the choroidal layer after the injection indicated successful delivery of clo-lip into the SCS. Tracer-based ocular cryosection analyses further showed robust fluorescence in the suprachoroidal region after SCS injection, whereas no appreciable fluorescence was observed in the neural retina, aqueous humor, and cornea, supporting that SCS delivery produces choroidal confined exposure rather than broad intraocular dissemination (Figure 2B). Based on these validations, mice were subjected to repeated SCS injections of PBS or clo-lip and subsequently analyzed by SD-OCT and choroidal assays to evaluate ocular outcomes (Figure 2C). Two weeks after treatment, the mice in the clo-lip group exhibited a myopic shift (−9.46 ± 3.76 D vs. +2.25 ± 2.89 D, *p* < 0.01), axial length elongation (0.45 ± 0.10 mm vs. 0.22 ± 0.04 mm, *p* < 0.001), and choroidal thinning (−7.10 ± 1.11 μm vs. 3.28 ± 1.97 μm, *p* < 0.001) compared with the controls (Figure S1C). Whole-mount choroidal immunostaining revealed a significant reduction in F4/80+ and IBA1+ cells (45.45% ± 8.15 vs. 1.08% ± 1.67, *p* < 0.001) in the clo-lip group (Figure 2E). These findings indicate that the localized depletion of choroidal macrophages after the injection of clo-lip into the SCS leads to choroidal thinning and myopia. To exclude potential developmental confounding, we repeated the clo-lip SCS injection in adult mice (12 weeks old). Adult mice received SCS injections of PBS or clo-lip using the same protocol as in juvenile mice. Two weeks after injection, clo-lip SCS injection induced a significant myopic refractive shift and axial length elongation compared with PBS-injected controls (Figure f and Figure S1D). Flow cytometric analysis further confirmed the effective depletion of choroidal macrophages in the clo-lip treated group (Figure S1E). These findings confirm that the myopia induced by choroidal macrophage depletion reflects an active pathological mechanism rather than a nonspecific disruption of normal ocular development.

### Reconstitution with polarized macrophages significantly modulated myopia progression in choroidal macrophage-depleted mice

**Figure 3:**
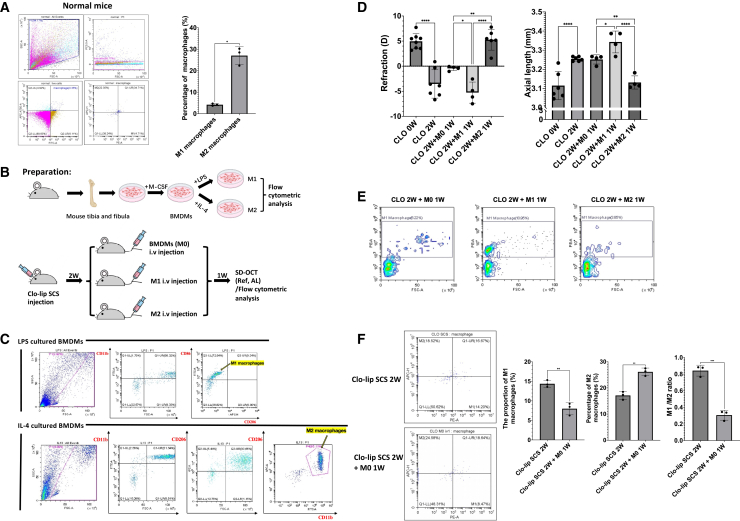
Macrophage polarization state determines susceptibility to myopia progression following choroidal macrophage depletion (A) Flow cytometric analysis of macrophage subsets in the choroid of normal mice. Quantification shows that M2 macrophages are significantly more numerous than M1 macrophages under physiological conditions (*p* < 0.05). Data are presented as mean ± SEM. Representative flow cytometry plots from three independent experiments are shown. (B) Schematic illustration of bone marrow-derived macrophage (BMDM) preparation, polarization, and adoptive transfer strategy. BMDMs were isolated from mouse tibia and fibula, differentiated with M-CSF, and polarized *in vitro* into M1 macrophages using LPS or into M2 macrophages using IL-4. Following two weeks of subconjunctival clodronate liposome (Clo-lip) injection to deplete endogenous choroidal macrophages, mice received intravenous injection of unpolarized (M0), M1, or M2 polarized BMDMs. Ref and AL were assessed one week later by SD-OCT, followed by flow cytometric analysis. Experiments were independently repeated three times with similar results. (C) Flow cytometry verified the successful polarization of bone marrow-derived macrophages into M1 macrophages (via LPS) or M2 macrophages (via IL-4/IL-13). (D) Mice that received M1-polarized macrophages exhibited an increased myopic shift (*p* < 0.05) and axial elongation (*p* < 0.05), whereas M2-polarized macrophages attenuated these changes (*p* < 0.0001). Bars represent mean ± SD. (Each data point represents one mouse; *n* = 4 per group). (E) Post-experimental flow cytometric analysis of choroidal tissues revealed distinct differences in M1 macrophage populations among the treatment groups. (F) Quantitative analysis of macrophage polarization in the choroid following adoptive transfer. Compared with Clo-lip treatment alone, M0 macrophage transfer significantly reduced the proportion of M1 macrophages (*p* < 0.01) and increased M2 macrophage levels (*p* < 0.01), resulting in a decreased M1/M2 ratio (*p* < 0.001). Statistical significance was assessed using two-way ANOVA with Bonferroni correction. *∗p < 0.05, ∗∗p < 0.01, ∗∗∗p < 0.001, and ∗∗∗∗p < 0.0001*.

In addition to depleting other mononuclear phagocytes, clo-lip also has a significant effect on the function of polymorphonuclear neutrophils.^37^ Therefore, macrophage-specific manipulation is required to rigorously determine the contribution of choroidal macrophages to myopia development. Before performing macrophage reconstitution experiments, we first characterized the baseline polarization status of choroidal macrophages under physiological conditions. Flow cytometric analysis of choroidal tissue from untreated, normal mice revealed that M2 macrophages constituted the dominant macrophage population, whereas M1 macrophages accounted for only a minor fraction (27.03% ± 4.14 vs. 4.18% ± 0.52, *p* < 0.05), indicating that the normal choroidal microenvironment is skewed toward a mildly anti-inflammatory, M2-dominant state (Figure 3A). In this protocol (Figure 3B), flow cytometry confirmed that these macrophages were successfully polarized into M1 (CD86^+^CD206^-^) and M2 (CD11b^+^CD206^+^) phenotypes *in vitro* before injection (Figure 3C). Two weeks after the clo-lip SCS injection, the macrophage-depleted mice exhibited a myopic shift (−3.53 ± 2.84 D vs. +4.96 ± 1.51 D, *p* < 0.0001) and axial elongation (3.255 ± 0.012 mm vs. 3.115 ± 0.073 mm, *p* < 0.0001). Subsequent tail intravenous administration of M1 macrophages further increased the myopic refractive shift (−5.27 ± 2.10 D vs. −0.44 ± 0.43 D, *p* < 0.05) and axial length (3.343 ± 0.057 mm vs. 3.252 ± 0.024 mm, *p* < 0.05) compared to M0-treated mice. In contrast, M2 macrophage reconstitution reversed the refractive error toward hyperopia (5.28 ± 2.08 D vs. −0.44 ± 0.43 D, *p* < 0.01) and reduced the axial length elongation (3.131 ± 0.035 mm vs. 3.252 ± 0.024 mm, *p* < 0.01) (Figure 3D). To assess macrophage polarization after reconstitution, we first examined changes in the M1 macrophage population. Flow cytometric analysis showed that M1 macrophages were increased in the M1-reconstituted group and reduced in the M2-reconstituted group (Figure 3E). Although M0 macrophages were not polarized prior to transfer, additional analysis revealed that M0 reconstitution significantly reduced the M1/M2 ratio in the choroid, indicating a relative shift toward an M2-like phenotype (Figure 3F). This shift provides a mechanistic explanation for the functional improvement observed after M0 reconstitution.

### Continuous injection of LPS stimulated choroidal M1 macrophage polarization and induced myopia in a murine model

**Figure 4:**
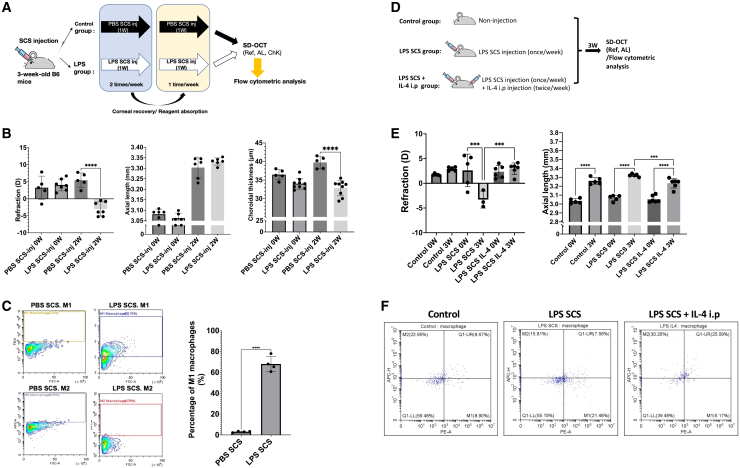
LPS-induced inflammatory polarization of choroidal macrophages promotes myopia progression and is reversed by IL-4 treatment (A) Experimental design for the SCS administration of LPS. Three-week-old C57BL/6 mice (*n* = 6 per group) received SCS injections of PBS or LPS following a defined dosing schedule that allowed corneal recovery and reagent absorption. Ref, AL, and ChK were assessed by SD-OCT, followed by flow cytometric analysis of choroidal macrophages. (B) Compared with PBS SCS injection, repeated LPS SCS injection induced a significant myopic shift (*p* < 0.0001) and choroidal thinning (*p* < 0.0001) after two weeks. Individual data points represent single eyes; bars indicate mean ± SEM. (C) Visualization of the flow cytometry results revealed a significant increase in M1 macrophages in the LPS SCS-injected group compared with the PBS SCS-injected group (*p* < 0.0001). Representative flow cytometry plots from two independent experiments are shown. (D) Experimental design (*n* = 5 per group). Mice received SCS LPS injections once weekly, with or without concurrent intraperitoneal (i.p.) IL-4 administration. Ocular biometry and flow cytometric analyses were performed after three weeks. (E) IL-4 administration significantly attenuated the myopic shift (*p* < 0.001) and axial elongation (*p* < 0.001) induced by LPS SCS injection. Data are presented as mean ± SEM. (F) Flow cytometric analysis of macrophage polarization following IL-4 treatment. Representative dot plots demonstrate that IL-4 administration reduced the proportion of M1 macrophages and increased M2 macrophages in the choroid of LPS-treated mice, indicating a shift toward an anti-inflammatory macrophage phenotype. Representative flow cytometry plots from three independent experiments are shown. *p* values were obtained using unpaired two-tailed t-tests and two-way ANOVA with Tukey post hoc. ∗*p* < 0.05, ∗∗*p* < 0.01, ∗∗∗*p* < 0.001, and ∗∗∗∗*p* < 0.0001.

To verify if the activation of M1 macrophages promotes myopia not only in the choroidal immune cell deficient mouse model but also in normal mice, we administered LPS to 3-week-old C57BL/6J mice in Figure S2A. After 24 h of treatment, real-time PCR revealed significantly upregulated *Nox2, Nox4, Mmp2,* and *Mmp9* mRNA expression in the LPS-injected group compared with PBS controls (Figure S2B). Consistently, ELISA demonstrated elevated levels of 8-hydroxy-2′-deoxyguanosine (8-OHdG) in choroidal tissue, indicating enhanced oxidative stress following LPS stimulation (Figure S2C). According to the experimental design (Figure S2D). One week after treatment, the LPS-injected mice exhibited a refractive shift (−9.21 ± 5.91 D vs. +2.91 ± 3.91 D, *p* < 0.001), an increased axial length/body weight ratio (0.28 ± 0.01 mm/g vs. 0.22 ± 0.005 mm/g, *p* < 0.0001), and choroidal thinning (−1.11 ± 0.90 μm vs. 1.36 ± 0.70 μm, *p* < 0.0001), compared to the PBS-injected mice. Notably, these changes persisted at 2 weeks post-injection (Figures S1E and S1F). To assess functional changes in choroidal vascular homeostasis following immune modulation, choroidal blood perfusion (ChBP) using optical coherence tomography angiography (OCTA), and the results indicated that the mice injected with LPS for 2 weeks showed reduced ChBP compared to the mice injected with PBS (37.36 ± 3.74% area vs. 46.79 ± 4.74% area, *p* < 0.001) (Figure S2G). While real-time PCR revealed the upregulation of the pro-inflammatory cytokines *Tnf-a* and *Il-6* in the LPS-treated mice (Figure S2H). Similarly, in this protocol (Figure 4A), SCS injection of LPS led to a comparable myopic refractive shift (−7.04 ± 1.92 D vs. +2.25 ± 2.89 D, *p* < 0.001), a greater increase in axial length (0.26 ± 0.017 mm vs. 0.22 ± 0.038 mm, *p* < 0.05), and significant choroidal thinning (−1.16 ± 1.88 μm vs. 3.28 ± 1.97 μm, *p* < 0.01) (Figure S3A). Flow cytometry of choroidal tissues revealed that LPS treatment significantly shifted macrophage polarization toward an M1 phenotype (CD45^+^F4/80^+^CD86^+^), with a corresponding decrease in M2 cells (CD45^+^F4/80^+^CD206^+^) (Figure 4C). These results imply that the SCS injection of LPS induced M1 macrophage polarization in choroidal macrophages, resulting in the development of myopia in mice. In addition to the myopic phenotype, LPS SCS injection also elicited a pronounced oxidative stress response in the choroid, as evidenced by increased 8-OHdG immunoreactivity (Figures S3B–S3D), further indicating the activation of an inflammatory microenvironment.

Given that the SCS injection of LPS established an M1-dominant inflammatory environment and induced myopia, we next examined whether shifting macrophage polarization toward an M2 phenotype could counteract LPS-induced myopia (Figure 4D). We found LPS SCS injection induced a significant myopic shift (−3.29 ± 3.82 D vs. +2.62 ± 3.15 D, *p* < 0.001) and axial length elongation (3.32 ± 0.021 mm vs. 3.07 ± 0.038 mm, *p* < 0.0001). Systemic administration of IL-4 markedly attenuated LPS-induced myopia, as evidenced by refractive error and axial length measurements (Figure 4E). Representative flow cytometry plots show that LPS SCS injection increased the proportion of M1-polarized macrophages, whereas systemic IL-4 administration shifted macrophage polarization toward an M2-dominant phenotype (Figure 4F). These results demonstrate that LPS-induced myopia can be attenuated by shifting the inflammatory environment.

To exclude the possibility that the observed refractive and axial length changes were secondary to generalized retinal dysfunction, we performed full-field ERG recordings in key experimental groups. SCS LPS injection resulted in significant suppression of both a- and b-wave amplitudes, which persisted during the experimental period (Figure S3E). Notably, despite persistent ERG suppression, systemic IL-4 administration markedly attenuated LPS-induced myopia. These findings indicate that the modulation of myopia by IL-4 occurs independently of ERG-detectable retinal dysfunction.

### Intraperitoneal and SCS injections of IL-4 inhibited the progression of myopia in a lens-induced myopia mouse model

**Figure 5:**
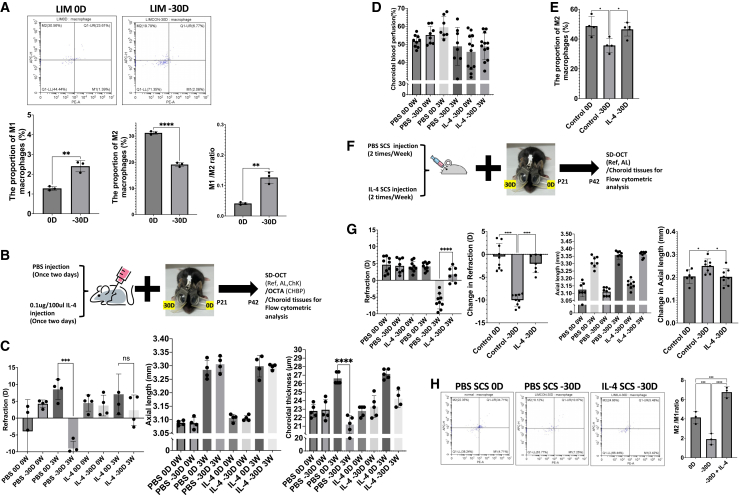
IL-4-mediated M2 macrophage polarization mitigated lens-induced myopia (A) Endogenous changes in choroidal macrophage polarization during lens-induced myopia (LIM). Flow cytometry quantification demonstrates a significant increase in the proportion of M1 macrophages (*p* < 0.01), a decrease in M2 macrophages (*p* < 0.0001), and an elevated M1/M2 ratio (*p* < 0.01) at LIM-30D compared with LIM-0D. Representative flow cytometry plots from three independent experiments are shown. Data are presented as mean ± SEM. (B) Experimental design for IL-4 intervention during LIM (*n* = 4 per group). Mice undergoing LIM received intraperitoneal injections of PBS or IL-4 (0.1 μg/100 μL) once every two days from P21 to P42. Ocular biometry was assessed by SD-OCT, choroidal blood perfusion was measured by OCTA, and choroidal tissues were collected for flow cytometric analysis. (C) IL-4 administration significantly attenuated the myopic shift and preserved choroidal thickness induced by LIM. Individual data points represent single eyes; bars indicate mean ± SEM. (D) Choroidal blood perfusion during LIM and IL-4 treatment. (E) Quantification of M2 macrophage proportion in the choroid during LIM. LIM-30D eyes exhibited a significant reduction in M2 macrophages (*p* < 0.05) compared with controls, whereas IL-4 treatment restored the proportion of M2 macrophages (*p* < 0.05). Data are presented as mean ± SEM. (F) Experimental scheme for the SCS administration of PBS or IL-4 during LIM. Mice received SCS injections twice per week during the LIM period, followed by SD-OCT measurements and flow cytometric analysis of choroidal tissues. (G) IL-4 SCS injection significantly suppressed LIM-induced myopic shift (*p* < 0.0001) compared with PBS SCS controls. Data are shown as mean ± SEM. (H) Flow cytometric analysis of macrophage polarization following SCS IL-4 treatment. Representative plots and quantification show that IL-4 SCS injection increased the M2/M1 ratio in the choroid compared with LIM-30D controls (*p* < 0.0001), indicating the restoration of an anti-inflammatory macrophage profile. Data are presented as mean ± SEM. *p* values were obtained using two-tailed t-tests and two-way ANOVA with Tukey HSD. ∗*p* < 0.05, ∗∗*p* < 0.01, ∗∗∗*p* < 0.001, and ∗∗∗∗*p* < 0.0001.

Given that the modulation of the inflammatory milieu attenuated LPS-induced myopia, we next examined choroidal macrophage polarization under physiological conditions and during lens-induced myopia (LIM). Flow cytometric analysis revealed that in LIM 0D, choroidal macrophages exhibited an M2-dominant polarization profile. In contrast, LIM -30D showed the proportion of M2-polarized macrophages was significantly reduced, accompanied by a relative increase in M1-polarized macrophages, resulting in a marked increase in the M1/M2 ratio (Figure 5A). These results indicate that myopia progression is associated with an endogenous shift of choroidal macrophages from an M2-dominant homeostatic state toward an M1-biased inflammatory phenotype. Given that myopia development is associated with an endogenous shift toward M1 macrophage polarization in the choroid, and that LPS-induced M1 polarization promotes myopia progression, driving choroidal macrophages toward an M2 phenotype is expected to exert a protective effect against myopia. In this protocol (Figure S4A). Flow cytometry results indicated a significant increase in the proportion of M2 macrophages from baseline to 48 h post-intraperitoneal IL-4 administration (Figure S4B), accompanied by upregulated *Cd206* and *Cd163* mRNA expression (Figure S4C). This finding suggested that injection every two days was an optimal regimen. Consistently, ELISA demonstrated a significant decrease in choroidal 8-OHdG levels in the IL-4-treated group compared to controls (Figure S4D), suggesting reduced oxidative stress. Furthermore, real-time PCR analysis revealed markedly reduced expression levels of *Nox2* and *Mmp9* mRNA in the choroid at 48 h after IL-4 injection (Figure S4E). Consistent with the endogenous polarization shift observed during myopia development, flow cytometric analysis revealed a significant increase in M1 macrophages, accompanied by a reduction in M2 macrophages, in the choroid following LIM (Figure 5A). Based on this observation, we next investigated whether promoting M2 macrophages polarization via alternate-day intraperitoneal IL-4 administration could attenuate myopia progression in a LIM mouse model (Figure 5B). In the PBS injection group, eyes treated with −30 D lenses showed a myopic shift in refractive status (−11.52 ± 4.91 D vs. +12.45 ± 8.19 D, *p* < 0.001), increased axial length (0.22 ± 0.01 mm vs. 0.18 ± 0.01 mm, *p* < 0.001), and decreased choroidal thickness (−1.74 ± 0.66 μm vs. 3.91 ± 0.95 μm, *p* < 0.0001) compared with the eyes treated with 0D lenses. In contrast, the eyes in the IL-4 injection group treated with −30D lenses showed smaller refractive change (−1.39 ± 2.91 D vs. −11.52 ± 4.91 D, *p* < 0.05), smaller axial elongation (0.19 ± 0.01 mm vs. 0.22 ± 0.01 mm, *p* < 0.01), and less thickening of the choroid (1.39 ± 0.19 μm vs. −1.74 ± 0.66 μm, *p* < 0.0001) than the eyes in the PBS-injected group with −30D lenses (Figure S4F). In addition, OCTA analysis showed that IL-4 treatment improved ChBP (1.54 ± 9.34% vs. −6.29 ± 8.73%, *p* < 0.05; Figure 5D). Furthermore, flow cytometry confirmed a higher M2 macrophage fraction in IL-4-treated mice than in PBS-treated mice (46.52 ± 0.05% vs. 35.71 ± 0.06%, *p* < 0.05; Figure 5E). IL-4 was administered to the SCS to evaluate its effect on choroidal macrophages. Western blot analysis revealed an increase in STAT6 phosphorylation at Tyr641 after 1 day of IL-4 SCS injection, indicating effective activation of the canonical IL-4 STAT6 signaling pathway in the choroid (Supplementary Figure g). In parallel, immunofluorescence staining for 8-OHdG demonstrated a marked reduction in oxidative DNA damage in the choroid following IL-4 SCS injection compared with controls (Supplementary Figure h-j), suggesting that IL-4 attenuates oxidative stress in the choroidal microenvironment. We next asked whether these molecular and microenvironmental changes translate into functional protection against myopia progression. To address this, we evaluated the effect of IL-4 SCS injection in a LIM model (Figure 5F). After the induction of myopia, the PBS-treated mice exhibited greater myopic shift (−10.18 ± 1.31 D vs. −0.55 ± 2.87 D, *p* < 0.0001) and greater changes in axial length elongation (0.25 ± 0.03 mm vs. 0.21 ± 0.03 mm, *p* < 0.05) than the IL-4 treated mice. In contrast, the myopia-induced mice that received IL-4 SCS injection showed less refractive change (−2.07 ± 1.98 D vs. −10.18 ± 1.31 D, *p* < 0.0001) and less axial length elongation (0.20 ± 0.04 mm vs. 0.25 ± 0.03 mm, *p* < 0.05) than the PBS-treated mice (Figure 5G). Flow cytometric analysis of choroidal macrophages showed that IL-4 SCS injection significantly increased the M2/M1 ratio under both physiological conditions (LIM 0D) and during LIM (LIM −30D) (Figure 5H), indicating a shift toward an M2-like macrophage phenotype in the choroid.

To reinforce the conclusion that M2 macrophages can inhibit myopia progression, we performed the same experiments using a 0.1 μg/100 μL IL-13 injection instead of an IL-4 injection and found that the IL-13 injection similarly promoted M2 polarization, suppressed myopia progression, and enhanced choroidal perfusion (Figures S5A–S5G). In addition to cytokine-based approaches, topical administration of berberine, a compound reported to promote M2-like macrophage polarization, significantly attenuated myopia progression in LIM mice, as reflected by reduced refractive error, axial elongation, and choroidal thinning (Figures S6A–S6C). Together with the functional rescue observed in the LPS SCS ± IL-4 model, these results support the notion that modulation of macrophage polarization state is closely associated with myopia outcomes *in vivo*.

## Discussion

Macrophages have been increasingly implicated in ocular growth regulation; however, whether choroidal macrophages play an important role in myopia development, and whether distinct polarization states exert differential effects, remains unclear. In the present study, we demonstrate that choroidal M1-polarized macrophages accelerate myopia progression, whereas M2-polarized macrophages suppress its development. To systematically address this issue, we sought to define the causal involvement and functional specificity of choroidal macrophages in myopia progression, informed by their established roles in regulating choroidal structure and tissue homeostasis.

To rigorously distinguish macrophage-specific effects from potential confounding influences of other immune cell populations, we employed a depletion-reconstitution strategy. Following SCS injection of clo-lip to broadly reduce local phagocytic myeloid cells, mice were selectively reconstituted via intravenous transfer of *in vitro*-polarized macrophages (M0, M1, or M2). Under conditions in which other clodronate-affected immune cells remained depleted, supplementation with macrophages of different polarization states produced a graded and directional functional outcome: M1 supplementation enhanced pro-inflammatory profiles and exacerbated myopia progression, M0 supplementation resulted in an intermediate effect, whereas M2 supplementation most effectively attenuated myopia development. These findings indicate that the initial polarization state of supplemented macrophages exerts a decisive influence on their *in vivo* functional impact, supporting the conclusion that macrophages are not only necessary but also sufficient to directionally modulate myopia progression in a polarization-dependent manner.

Resident macrophages are densely distributed within the perivascular space of the choroid, where they exhibit a high degree of specialization, playing a pivotal role in maintaining choroidal vascular homeostasis and orchestrating inflammatory responses to pathological insults.^14^^,^^16^^,^^38^^,^^39^ This vascular homeostatic balance is not only crucial for immune regulation but has also been closely linked to the preservation of choroidal thickness, a key anatomical parameter inversely correlated with myopia progression.^24^^,^^40^ The disruption of choroidal vascular homeostasis, as seen in various pathological conditions, is often accompanied by choroidal thinning and has been implicated in the development of axial elongation.^26^ Because oxidative stress is a well-recognized contributor to choroidal dysfunction and axial elongation,^41^^,^^42^^,^^43^ and because it is not merely a consequence of tissue injury but is actively regulated by the polarization state of choroidal macrophages,^44^^,^^45^ we next examined whether macrophage polarization modulates oxidative stress-related pathways in the choroid. Our results indicate that M1-polarized macrophages are associated with increased oxidative damage, as evidenced by the elevated expression of NADPH oxidase components (Nox2, Nox4), matrix metalloproteinases (Mmp2, Mmp9), and increased levels of the oxidative DNA damage marker 8-OHdG. In addition to ELISA measurements, immunohistochemical analysis further revealed increased 8-OHdG immunoreactivity following M1 macrophage activation, with signals detected across ocular tissues and in the retina-RPE-choroid region. In contrast, M2 macrophage polarization was associated with reduced oxidative stress markers and attenuated 8-OHdG signals. Functionally, M1 activation led to choroidal thinning and promoted myopia progression, whereas M2 polarization preserved choroidal thickness and attenuated myopic development. Together, these findings support a close association between macrophage polarization, oxidative stress, and choroidal homeostasis in myopia.

In addition to regulating oxidative stress, macrophage polarization also critically modulates local inflammatory responses. M1 macrophages produce pro-inflammatory cytokines, leading to tissue remodeling and disease progression. Ocular inflammatory diseases, such as allergic conjunctivitis, juvenile chronic arthritis-related uveitis, and multifocal choroiditis, are associated with an increased risk of myopia and elevated levels of inflammatory cytokines.^26^^,^^46^^,^^47^ To determine whether macrophage-driven inflammation actively contributes to myopia rather than reflecting a secondary response, we experimentally manipulated macrophage polarization states *in vivo* using LPS-induced M1 activation and complementary M2-polarizing interventions. Consistent with the known role of macrophage polarization in regulating local inflammation, our findings provide further evidence linking M1 macrophage activation to myopia development. Specifically, we observed a marked increase in the expression of M1-associated markers in the choroid of mice with LIM. Furthermore, experimental induction of M1 polarization in normal mice led to elevated levels of pro-inflammatory cytokines (TNF-α and IL-6) and subsequently promoted myopia onset. These results suggest that M1-polarized macrophage responses may contribute to an inflammatory microenvironment that facilitates choroidal thickness thinning and axial length elongation. Although SCS LPS induced measurable suppression of ERG responses, the dissociation between persistent ERG deficits and improved myopic outcomes indicates that choroidal macrophage immunomodulation can regulate myopia progression independently of gross retinal dysfunction. In contrast, M2 macrophages exert anti-inflammatory effects by releasing cytokines such as IL-10 and TGF-β, promoting tissue repair and immunoregulation. Emerging evidence suggests that several compounds with the potential to suppress myopia progression also exhibit anti-inflammatory properties. Resveratrol eye drops prevented inflammation and myopia progression in a form-deprivation myopia (FDM) model.^48^ Orally administered lactoferrin (LF), which also exhibits anti-inflammatory effects, reduced myopia in a LIM mouse model.^49^ However, the specific role of anti-inflammatory macrophages in myopia remains unclear. Both cytokine-based and pharmacological approaches were employed to modulate M2 macrophage polarization, allowing us to test whether the observed effects on myopia were robust across mechanistically distinct interventions. In the present study, intravenous administration of IL-4-polarized M2 macrophages significantly mitigated myopia progression in choroidal macrophage-depleted mice. Additionally, injections of IL-4 and IL-13, known to induce M2 macrophage polarization, activated anti-inflammatory responses, increased choroidal thickness, and improved blood perfusion. Consistent with prior *in vivo* studies, dosing frequency was guided by established experimental protocols and functional readouts of biological activity.^50^^,^^51^ These findings suggest that therapeutic strategies leveraging compounds such as berberine, which in our study promoted M2 macrophage polarization, may represent a potential approach for controlling myopia progression, particularly in cases characterized by inflammation-driven choroidal dysfunction. While our study does not delineate all downstream molecular mediators, the convergence of loss-of-function, gain-of-function, and rescue approaches supports a direct pathogenic role of choroidal macrophage polarization in myopia. In this context, while scleral extracellular matrix remodeling represents the ultimate structural determinant of axial elongation, the present study was designed to interrogate an upstream regulatory layer of myopia progression, choroidal immune and vascular homeostasis, rather than to directly characterize downstream scleral extracellular matrix changes. Elucidating the molecular pathways linking choroidal immune regulation to scleral fibroblast activity and matrix remodeling will be an important focus of future studies.

Under physiological conditions, M2 macrophages protect the endothelial barrier and facilitate vascular stability and maturation.^52^^,^^53^ In pathological settings, the attenuation of macrophage-driven inflammation or polarization toward the M2 phenotype has been shown to prevent intracranial aneurysm rupture^54^ and facilitate aortic wall healing by preserving vascular integrity during disease progression.^55^ These findings underscore the capacity of M2 macrophages to maintain vascular homeostasis, supporting their potential role in stabilizing the choroidal vasculature and mitigating myopia progression. Although a previous study demonstrated the polarization of M2 macrophages in the sclera of an FDM model,^56^ that research primarily focused on extracellular matrix remodeling and did not address the role of choroidal macrophages. In addition, it should be noted that macrophage mechanisms differ in different tissues. Macrophages interact with fibroblasts in the sclera to maintain scleral morphology. However, they are more abundant in the choroid than in the sclera and play an important role in choroidal vascular homeostasis. Taken together with our experimental findings, myopia development is accompanied by an endogenous shift of choroidal macrophages toward an M1-polarized state, and LPS-induced M1 polarization further accelerates myopia progression. In contrast, interventions that drive choroidal macrophages toward an M2 phenotype exert a protective effect against myopia. In this context, our findings suggest that choroidal M2 macrophages contribute to maintaining choroidal vascular stability and suppressing myopia progression through anti-inflammatory and vascular maintenance mechanisms. These findings suggest that tissue-specific macrophage polarization may differentially impact ocular structures during myopia progression and may inform future studies of immunomodulatory targets for therapeutic intervention.

In conclusion, the results of this study indicate that macrophage polarization is a factor that affects the progression of myopia, primarily through inflammation, vascular stabilization, and modulation of oxidative stress. These findings suggest that the regulation of macrophage phenotypes may be relevant to therapeutic strategies for myopia.

### Limitations of the study

This study has several limitations. Although multiple complementary approaches consistently supported a role for choroidal macrophage polarization in myopia progression, the experiments were performed in mouse models, and caution is needed when extrapolating these findings to human myopia. Moreover, only male mice were used in this study, and potential sex-dependent differences in macrophage polarization and myopia progression were not evaluated. While our results suggest that macrophage polarization influences myopia through the regulation of choroidal inflammation, oxidative stress, and vascular homeostasis, the downstream molecular mechanisms linking these changes to scleral remodeling and axial elongation were not fully resolved. Therefore, additional studies are warranted to define these mechanisms more precisely and to establish their relevance to human myopia.

## Resource availability

### Lead contact

For further information or resource requests, please contact the lead contact, Toshihide Kurihara (kurihara.z8@keio.jp).

### Materials availability

This study did not generate new unique reagents.

### Data and code availability

All data reported in this paper are included in the main text, figures, and supplemental information. This study did not generate new unique reagents. Any additional information required to reanalyze the data reported in this paper is available from the lead contact upon reasonable request.

## Acknowledgments

The authors thank Y. Katada, N. Ban, A. Nakai, N. Serizawa, C. Shoda, M. Ziyan, C. Junhan, and A. Kawabata (Graduate School of Medicine, Keio University, Tokyo, Japan) for their technical guidance and administrative support. This work is supported by 10.13039/100009619AMED under grant no. JP22gm1510007 to T. Kurihara.

## Author contributions

Conceptualization, J.H., S.I., T.K., and K.T.; methodology, J.H. and S.I.; formal analysis, J.H.; investigation, J.H.; data curation, J.H.; project administration, J.H., S.I. and T. K.; writing – original draft Preparation, J.H.; writing – review and editing, S.I., K.M., J.H., H.T., K.N., T.K., and K.T.; supervision, K.N., T.K., and K.T. All authors made a substantial contribution in the revision of the manuscript.

## Declaration of interests

K.T. reports that he is the CEO of Tsubota Laboratory, Inc., Tokyo, Japan, a company that develops products for the treatment of myopia. The other authors have declared no conflicts of interest. Patent-pending (J.H. S.I. T.K. K.T. 2022–72596).

## STAR★Methods

### Key resources table

**Table:** 

REAGENT or RESOURCE	SOURCE	IDENTIFIERS/ADDITIONAL INFORMATION
**Antibodies**		

Anti-F4/80 antibody [EPR26545-166]	Abcam	Catalog#ab300421; RRID: AB_2924916
Anti-Iba1 antibody [EPR16588]	Abcam	Catalog#ab289874; RRID: AB_2861174
7-AAD Viability Staining Solution	eBioscience	Catalog#00-6993-50
PB450 Anti-mouse CD45	Invitrogen	Catalog#48-0451-82; RRID: AB_1518806
APC/Cy7 anti-mouse F4/80	BioLegend	Catalog#123117; RRID: AB_893481
FITC anti-mouse CD11b	BioLegend	Catalog#101205; RRID: AB_312788
PE Rat Anti-Mouse CD86	BD Pharmingen™	Catalog# 553692; RRID: AB_394993
CD206 (MMR) Monoclonal Antibody (MR6F3), APC,	ThermoFisher	Catalog#17-2061-82; RRID: AB_11149698

**Chemicals, peptides and recombinant proteins**		

Clodronate liposome	Liposoma BV	Catalog#29855
Recombinant Murine IL-4	PEPROTECH	Catalog# 214-14
Recombinant Murine IL-13	PEPROTECH	Catalog#210-13
DMEM (high glucose)	Gibco	Catalog#5796
FBS	Biowest	Catalog#S1650
Penicillin streptomycin	Gibco	Catalog#10378016
FITC-dextran 70 100 MG	TdB Labs AB	FD70
Anti-8-hydroxy-2′-deoxyguanosine (8-OHdG) antibody	Nikken	Catalog#30315331
Recombinant mouse CSF-1	R&D Systems	Catalog# 416-ML/CF

**Sequence-based reagent**		

mouse Il6 forward:	IDT	CTACCCCAATTTCCAATGCT
mouse Il6 reverse:	IDT	ACCACAGTGAGGAATGTCCA
mouse Tnfa forward:	IDT	CTGTAGCCCACGTCGTAGC
mouse Tnfa reverse:	IDT	TTGAGATCCATGCCGTTG
mouse Mrc1 forward:	IDT	TCGAGACTGCTGCTGAGTCCA
mouse Mrc1 reverse:	IDT	AGACAGGATTGTCGTTCAACCAAAG
mouse Nox2 forward:	IDT	ACTCCTTGGAGCACTGG
mouse Nox2 reverse:	IDT	GTTCCTGTCCAGTTGTCTTCG
mouse Nox4 forward:	IDT	TGAACTACAGTGAAGATTTCCTTGAAC
mouse Nox4 reverse:	IDT	GACACCCGTCAGACCAGGAA
mouse Mmp2 forward:	IDT	CAAGTTCCCCGGCGAT
mouse Mmp2 reverse:	IDT	TTCTGGTCAAGGTCAC
mouse Mmp9 forward:	IDT	GGACCCGAAGCGGACA
mouse Mmp9 reverse:	IDT	CGTCGTCGAAATGGGC
mouse GAPDH forward:	IDT	AGGAGCGAGACCCCACTAAC
mouse GAPDH reverse:	IDT	GATGACCCTTTTGGCTCCAC

**Mice**		

C57BL/6J mice	CLEA Japan, Inc.	N/A
BALB/cJ mice	CLEA Japan, Inc.	N/A

**Software**		

GraphPad Prism	GraphPad Software	RRID:SCR_002798
ImageJ	NIH (National Institutes of Health)	RRID:SCR_003070
CytExpert	Beckman Coulter Life Sciences	RRID:SCR_017217

### Experimental model and study participant details

#### Animals

Wild-type male C57BL/6J and BALB/cJ mice were used in this study. Juvenile mice were used from postnatal day 21 (P21), adult mice were used at P56 or 12 weeks of age depending on the experimental design, and 6–12-week-old male C57BL/6J mice were used as bone marrow donors for bone marrow-derived macrophage preparation. C57BL/6J mice were used for intraperitoneal injection, lens-induced myopia, and immunomodulation experiments, whereas BALB/cJ mice were used for selected suprachoroidal space injection experiments.

#### Animal care and study approval

All animal experiments were approved by the Institutional Animal Care and Use Committee of Keio University (approval number: 16017) and conducted in accordance with the Association for Research in Vision and Ophthalmology Statement for the Use of Animals in Ophthalmic and Vision Research, Keio University’s Institutional Guidelines on Animal Experimentation, the National Institutes of Health Guidelines for the Care and Use of Laboratory Animals, and the ARRIVE guidelines. Mice were housed in pathogen-free facilities under a 12:12-h light-dark cycle at 23 ± 3 °C with approximately 50 lux of fluorescent light (color temperature: 5000 K), in standard transparent cages (29 × 18 × 13 cm, 4–5 mice per cage), with free access to chow and water. Animals were randomly assigned to experimental groups.

#### Sex as a biological variable

Only male mice were used in this study. This decision was based on established methodological considerations for the lens-induced myopia (LIM) mouse model, which has been predominantly validated in males. Male mice exhibit more stable head size and body weight during the experimental period, allowing more reliable attachment and retention of the lens apparatus. In addition, the use of male mice minimizes potential confounding effects of sex hormones, which are known to influence ocular growth and immune responses. Previous work demonstrated that sex hormones play a regulatory role in myopia development in female mice,^57^ highlighting the potential for hormonal fluctuations to confound mechanistic analyses. Consistent with previous LIM studies, male mice were therefore selected to ensure experimental stability and mechanistic clarity. However, this study is limited by the use of only male animals, and the findings may not be fully generalizable to female mice. Future studies including both sexes will be needed to assess potential sex-dependent differences.

### Method details

#### Macrophage depletion mouse model

Intraperitoneal clodronate liposome (Liposoma BV, Amsterdam, The Netherlands) injection (dose: 0.10 mL/10 g) was administered to C57BL/6J mice (from P21 to P28 or from P56 to P63) once every two days. The control group was injected with an equal amount of PBS liposomes as described above. Refractive status, axial length, and choroidal thickness were measured before and after four injections. The mice were then euthanized, and their choroid samples were collected. In addition, following previously established protocols,^58^ 3 μL of clodronate liposome was injected into the suprachoroidal space (SCS) of P21 BALB/cJ mice once every two days to observe its effect on choroidal macrophage depletion.

#### Measurement of refractive status, axial length, and choroidal thickness

Refraction was measured using an infrared photorefractor (Steinbeis Transfer Center, Stuttgart, Baden-Württemberg, Germany) as previously reported.^59^ After measuring refraction, axial length, and choroidal thickness were measured using an SD-OCT system. For each mouse, measurements of refraction, axial length, and choroidal thickness were performed before the start of LIM (0W) and at the end of LIM (3W). Before the measurements, the mouse’s eyes were treated with mydriasis eye drops containing 0.5% tropicamide and 0.5% phenylephrine (Santen Pharmaceutical Co., Ltd., Osaka, Japan) to ensure mydriasis and cycloplegia. After pupil dilation, the mice were subjected to general anesthesia using MMB. It is important to prevent the occurrence of corneal injury during measurement. Axial length was measured as the vertical distance from the anterior corneal surface to the retinal pigment epithelium layer near the optic nerve.^59^ Choroidal thickness was determined by quantifying the circular area of the disc on the posterior surface of the choroid using ImageJ software.^60^

Because clodronate liposome treatment and LPS administration induced significant body weight reduction in specific experimental settings, and body weight is known to strongly influence ocular axial length in mice, axial length data from these 2 experiments were normalized to body weight (axial length/body weight, AL/BW) to minimize confounding effects. In experimental settings where body weight did not differ significantly between groups, absolute axial length values were used.

#### Flow cytometry

The mice were injected with an overdose of MMB to induce profound anesthesia before being euthanized via cervical dislocation. Their eyes were immediately enucleated and the anterior segment, vitreous, and retina were discarded. Choroidal tissues were carefully scraped off the sclera–choroid complex, collected in a tube (four choroid tissues/tube), and processed in a digestion buffer, which included 100 μg/mL Liberase (Sigma Aldrich Japan ROCHE), 25U/mL dispase (Thermo Fisher Scientific, Waltham, MA, USA), and 1U/mL DNase I (Worthington Biochemical, New Jersey, USA), for 40 min at 37°C. The choroid tissue was thoroughly stirred with a pipette every 5 min to disperse the choroidal cells. The digested choroid mixture was transferred into cell strainers, and any undigested choroid pieces were mashed using the end of a syringe plunger while adding 20 mL PBS solution containing 2% FBS to allow for the cells to pass through into new 50 mL tubes placed below the strainers. The aggregated choroidal cells were dissociated into single-cell suspensions via differential centrifugation. The supernatant was removed and the cells were washed by adding 3 mL red blood cell lysis buffer (Funakoshi, Tokyo, Japan) to the cell suspension. Subsequently, the cells were centrifuged several times and suspended in a PBS solution containing 2% FBS. The cell count was measured to determine whether the number of cells was sufficient for detection using flow cytometry. After the cells were counted, the cell suspension was replaced with 1% BSA and the cells were washed twice, stained with a mixture of fluorochrome-conjugated antibodies, including 1:200 PB450 Anti-mouse CD45 (Invitrogen, Waltham, MA, USA), 1:200 FITC anti-mouse CD11b M1/70 (BioLegend, California, USA), 1:200 APC/Cyanine7 anti-mouse F4/80 (BioLegend, California, USA), 1:200 CD206 (MMR) Monoclonal Antibody (MR6F3) APC (ThermoFisher, Waltham, MA, USA), and 1:200 PE Rat Anti-Mouse CD86, BD (Pharmingen,NJ, USA), for at least 30 min and covered with 1 μL 7-AAD (eBioscience, San Diego, USA) for 5 min at room temperature. All incubations without 7-AAD were performed on ice and light was avoided. Data were acquired using a CytoFLEX S flow cytometer and the CytExpert software (Beckman Coulter Life Sciences, Inc., Indiana, United States). Offline data analysis was performed using the CytExpert software. All quantitative results were derived directly from raw flow cytometry events obtained from individual choroidal samples using a predefined and consistent gating strategy. The same marker combinations and gating criteria were applied across all experiments in this study. Representative gating strategies are shown in other figures.

#### Suprachoroidal space injections

Mice were anesthetized with a mixture of midazolam (Sandoz K.K., Tokyo, Japan), medetomidine (Domitor, Orion Corporation, Espoo, Finland), and butorphanol tartrate (MMB) (Meiji Seika Pharma Co., Ltd., Tokyo, Japan) and taped to be mounted under a microscope for evaluation of the eyes and injections. A 5 μL syringe (Super elastic plunger models – MS-E series, ITO Corporation, Shizuoka, Japan) with an extremely fine 0.15 mm needle (Special Interchangeable Needle SUS316L, ITO Corporation, Shizuoka, Japan), filled with 3 μL of a reagent, was inserted into the sclera at 1 mm behind the corneal limbus with the bevel facing down, slowly passing through the scleral fibers into the SCS. The syringe plunger was pushed slowly to expand the SCS, and 3 μL of the reagent was injected. The needle was held steady for about 30 s to ensure that all the contents of the syringe were injected and the SCS was fully expanded. Finally, the needle was pulled out and a cotton swab was gently placed on the injection site. For tracer experiments assessing the spatial distribution of substances delivered via SCS injection, fluorescein isothiocyanate–conjugated dextran (FITC–dextran, 70 kDa, 2 mg/mL in PBS; TdB Labs) was injected using the same procedure.

Using an SD-OCT system (Envisu R4310; Leica Microsystems, Wetzlar, Germany), the scleral choroidal layer was observed to confirm the presence of a shallow choroidal detachment on the injection side, indicating that the suprachoroidal injection was successfully completed. To verify the relationship between SCS injection and the development of myopia, the injection was administered twice in the first week to give the mice enough time to absorb the reagent and recover from any corneal damage. The injection frequency was adjusted to once a week in the second week.

#### Hematoxylin and eosin (HE) staining

To verify the success of the SCS injection, the eyeballs of the mice were enucleated within 2 h after the injection, collected, and fixed in an eye-specific fixative solution for 24 h. The ocular tissues were then dehydrated using a graded ethanol series, embedded in paraffin, and routine cut into 4 μm sections. The sections were stained with HE and mounted with a neutral resin for examination. PBS SCS injection was performed to verify the accuracy of the drug entry point. The eyeballs were enucleated within 2 h after SCS injection for paraffin sectioning and HE staining.

#### Immunohistochemical analysis of sclerochoroidal flat-mount

After several SCS injections of clodronate liposome/PBS liposome, the BALB/cJ mice were euthanized, their eyeballs were enucleated, and the anterior segment tissues were carefully removed. The sclerochoroidal eyecups were cut into four sections and fixed in 4% paraformaldehyde in PBS for 1 h at room temperature. Subsequently, sclerochoroidal whole-mounts were transferred to a blocker buffer (1 × PBS containing 5% bovine serum albumin (BSA) and 1% Triton X-100) for 1 h at room temperature. To process the tissue samples, the whole-mounts were first treated with a blocking buffer containing primary antibodies, which included F4/80 (1:100∼1:200; Abcam) and IBA-1 (1:100-1:200; Abcam), incubated at 4°C for 24 h. After washing the whole-mounts with blocking buffer three times for 15 min, secondary antibodies (Alexa Fluor 488- and 555- conjugated anti-rabbit and goat IgG) were applied for 6 h at 4°C to label specific proteins. A fluorescence microscope (Keyence, BZ-X800) was used to capture images of the stained samples. Tiled images of the sclerochoroidal whole-mounts were acquired with a 2x and 10× objective lens for analysis of macrophage distribution across the choroid. z stack was used to obtain distinct images for subsequent computer-assisted analyses (ImageJ 1.53a, NIH). The density of the macrophages throughout the entire choroid was quantified and represented as the number of cells per specified unit area.

#### Isolation and cultivation of bone marrow derived-macrophages

Bone marrow cells were harvested from male C57BL/6 mice aged between 6 and 12 weeks as reported.^61^ The mice were euthanized via cervical dislocation, and their femurs and tibiae were collected. The epiphyses of the femurs and tibiae were removed and each bone was flushed with 3 mL of sterile 1× PBS to extract the bone marrow. The bone marrow was suspended in bone marrow culture medium (high glucose DMEM, 10% fetal bovine serum [FBS], 1% penicillin streptomycin and 1 ng/ml M-CSF1) and mechanically dissociated and filtered through a 100 μm cell strainer to obtain single-cell suspensions. The cells were washed, resuspended in bone marrow culture medium, and transferred into a six-well plate at a concentration of 1×10^6^ cells/mL. Thereafter, the cultures were incubated at 37°C in a 5% CO_2_ atmosphere. On day 4, 1 mL/well of bone marrow culture medium was added and cultured until day 6. The resulting cells, referred to as M0 BMDMs, were not exposed to any additional polarizing agents and were used as baseline, non-polarized controls.

#### M1 and M2 macrophages polarization *in vitro*

After six days of differentiation, the bone marrow derived-macrophages were rinsed twice with sterile PBS as reported.^62^ To initiate M1 activation, 2 mL of DMEM containing 10% FBS and 1 μg/mL LPS were added to each well. For M2 activation, the medium was supplemented with 40 ng/mL IL-13 and 40 ng/mL IL-4 instead of LPS. The cells were then incubated for 24 h. The culture samples were analyzed using flow cytometry with CD86 to confirm M1 macrophage polarization and CD206 to verify M2 macrophage polarization.

#### Pharmacological and immunomodulatory interventions

To modulate macrophage polarization and evaluate its effect on myopia progression, mice were subjected to various systemic and local treatments. For M1 polarization, lipopolysaccharides (LPS) from *Escherichia coli* O111:B4 (Merck, Tokyo, Japan) were dissolved in PBS at 10 mg/mL and injected intraperitoneally at a dose of 10 μg/g body weight. Injections were adjusted according to individual mouse weight and administered under close monitoring to avoid excessive weight loss. PBS was used as the vehicle control. For M2 polarization, recombinant murine IL-4 (214-14) and IL-13 (210-13) (PeproTech, New Jersey, USA) were dissolved in PBS at 0.1 μg/100 μL and administered intraperitoneally every other day during the period of myopia induction. Control animals received an equal volume of PBS. Mouse body weight was recorded prior to each injection. Both eyes were injected. To account for inter-eye correlation, the mouse was used as the experimental unit; values from both eyes were averaged for statistical analysis.

For local ocular delivery of an M2-polarizing agent, berberine chloride hydrate (TCI, Tokyo, Japan) was freshly dissolved in 2% DMSO to yield a final concentration of 0.26 mg/mL. Given the limited aqueous solubility of berberine and potential cytotoxicity of DMSO, a 2% DMSO vehicle control group and an untreated control group were included to assess solvent effects.

#### Real-time quantitative PCR (qPCR)

After the mice were euthanized, their eyeballs were enucleated immediately to collect retinal, choroidal, and scleral tissues. The tissues were frozen in liquid nitrogen and stored at −80 °C for later use. Mouse total RNA was extracted from the retinal, choroidal, and scleral tissues using TRIzol (TRI reagent) (MOR, Miami, FL, USA #TR118). Small RNAs were isolated using RWT (QIAGEN, Hilden, Germany, #1067933) and RPE buffer (QIAGEN, Hilden, Germany, #1018013). The RNA samples were dissolved in RNase-free water (TAKARA HOLDINGS Inc., Kyoto, Japan, 9012) and measured using a spectrophotometer (NanoDrop; Thermo Fisher Scientific, Waltham, MA, USA). The extracted RNA was converted into cDNA after RNA denaturation, DNase reaction, and reverse transcription, according to the manufacturer’s instructions. mRNA gene expression was determined using SYBR Green RT-PCR with the cDNA template. In addition, PCR was performed using StepOnePlus Real-Time PCR System (Applied Biosystems, Waltham, Massachusetts, USA). mRNA expression of each gene was evaluated using the comparative Ct (ΔΔCT) method and normalized to glyceraldehyde-3-phosphate dehydrogenase mRNA expression, which was used as the reference gene.

#### 8-OHdG ELISA

For the assessment of oxidative stress, the level of 8-OHdG in the choroidal tissues was measured using commercial ELISA kits according to the manufacturer’s instructions (Uscn Life Science; Wuhan, China). As this assay uses a competitive inhibition enzyme immunoassay approach, the concentration of 8-OHdG in the sample and the assay signal strength are inversely correlated. The concentration of 8-OHdG in each sample was calculated using standard curves generated from standard proteins, which were calculated and analyzed using Boster’s ELISA Online Calculator.

#### Measurements of choroidal blood perfusion

Choroidal blood perfusion was measured at the beginning (3-week-old) and end (6-week-old) stages of myopia induction using an SS-OCT/OCTA device (XEPHILIO OCT-S1, CANON Medical Systems, Tokyo, Japan). The measurement method has been described in a previous report.^63^The measurement method has been described in a previous report.^63^ As with the refraction measurements, the pupils of the mice were dilated under general anesthesia before measurement. Choroidal blood perfusion was measured using *en face* angiography, with the optic nerve identified as the central area. The choroidal blood perfusion signals were identified using the B-scan images of the corresponding positions. The areas in the B-scan images covered by red noise points were the areas without blood perfusion. ImageJ quantitative analysis was used to determine the non-perfused areas in the choroid and evaluate the percentage of the perfused areas.

#### Electroretinography (ERG)

Full-field ERG recordings were performed in selected SCS-treated groups to assess whether refractive and axial length changes could be attributed to gross retinal dysfunction. Following an established protocol.^64^ Prior to measurement, mice were dark-adapted for 24 h, after which pupil dilation was induced and general anesthesia was administered via intraperitoneal injection of MMB. ERG signals were recorded using a PuREC system (MAYO, Inazawa, Japan), with recording electrodes positioned on the corneal surface. Responses were elicited using flash stimuli with intensities ranging from 0.02 to 10 cd s/m^2^. The a-wave amplitude was defined as the difference between the baseline and the most negative trough, while the b-wave amplitude was measured from the a-wave trough to the peak of the subsequent positive deflection. During recordings, mice were maintained on a temperature-controlled heating pad to preserve stable body temperature. All procedures were performed under dim red illumination to minimize photoreceptor bleaching.

#### Western blotting

Following an established protocol.^65^Choroidal tissues were collected at the indicated time points after treatment and homogenized in RIPA lysis buffer supplemented with protease and phosphatase inhibitors. Protein concentrations were determined using a BCA protein assay. Equal amounts of protein (7.5–10 μg per lane) were separated by SDS–PAGE on 10% polyacrylamide gels and transferred onto PVDF membranes (Merck Millipore, MA, USA).

Membranes were blocked with Blocking One (Nacalai Tesque, Tokyo, Japan) and incubated overnight at 4 °C with primary antibodies against phosphorylated STAT6 (Tyr641) (1:1000), total STAT6 (1:1000), and β-actin (1:5000) as a loading control. After washing, membranes were incubated with appropriate HRP-conjugated secondary antibodies (1:10,000) and visualized using SuperSignal West Femto Maximum Sensitivity Substrate (Thermo Fisher Scientific).

Protein bands were detected using a chemiluminescence imaging system, and densitometric analysis was performed using ImageJ software (version 1.53). Phosphorylated STAT6 levels were normalized to total STAT6, and protein expression levels were normalized to β-actin.

#### LIM mouse model

A murine LIM model was created as previously described.^59^ The mice were placed under general anesthesia using MMB. Spectacle frames that fit the head contours of the mice were designed and printed using a three-dimensional printer. For the induction of myopia, a −30D lens was constructed using polymethyl methacrylate. The left and right eye frames of the spectacles were adjusted to fit the shape of each mouse’s skull and screwed onto a stick. Each spectacle was fitted with a joint that enabled alteration or removal of the left and right frames for cleaning. Subsequently, the stick was bonded to the skull using a self-curing dental adhesive system. Induction of myopia began once the mouse had fully recovered from the anesthesia. The lenses were removed for cleaning at least twice a week.

### Quantification and statistical analyses

Statistical analyses were performed using GraphPad Prism, Prism 10 for macOS (GraphPad Software, San Diego, CA, USA). Data are presented as mean ± SEM. Comparisons between two groups were analyzed using unpaired two-tailed t-tests or Welch’s t-tests when variances were unequal. Comparisons among multiple groups were performed using one-way or two-way analysis of variance (ANOVA), followed by Tukey’s or Bonferroni post hoc tests as appropriate. A value of *p* < 0.05 was considered statistically significant. ∗*p* < 0.05. ∗∗*p* < 0.01. ∗∗∗*p* < 0.001. ∗∗∗∗*p* < 0.0001. Sample sizes (n), which represent the number of animals included in each analysis, and the specific statistical tests used are specified in the corresponding figure legends. All experiments were independently repeated at least twice to ensure reproducibility.

Due to technical limitations inherent to *in vivo* ocular measurements, the number of animals included in each analysis may vary slightly between parameters. In some cases, reliable measurements could not be obtained because of corneal injury, corneal opacity, suboptimal optical alignment, or motion artifacts during imaging. Only measurements meeting predefined quality criteria were included for statistical analysis, and exclusions were applied in a parameter-specific manner without reference to experimental outcome.

## Supplemental information

Document S1. Figures S1–S6

Data S1. Raw numerical data underlying all figures in this study
