# Supplementary material for: The choroidal macrophage polarization significantly influences myopia development in murine models
Source: iScience. 2026 Apr 17;29(5):115764. doi: 10.1016/j.isci.2026.115764 (PMC13157010; doi:10.1016/j.isci.2026.115764)
Supplement: Document S1. Figures S1–S6 [file mmc1.pdf]

iScience, Volume *29*

Supplemental information

The choroidal macrophage polarization significantly influences myopia development in murine models

Jing Hou, Shin-ichi Ikeda, Yajing Yang, Tomokazu Fukuchi, Chiaki Ikeda, Satoshi Imanishi, Ziyan Ma, Junhan Chen, Kiwako Mori, Hidemasa Torii, Hideki Fujii, Kazuno Negishi, Kazuo Tsubota, and Toshihide Kurihara

Supplementary Figure 1. Quantitative changes in ocular parameters following macrophage depletion in juvenile and adult mice

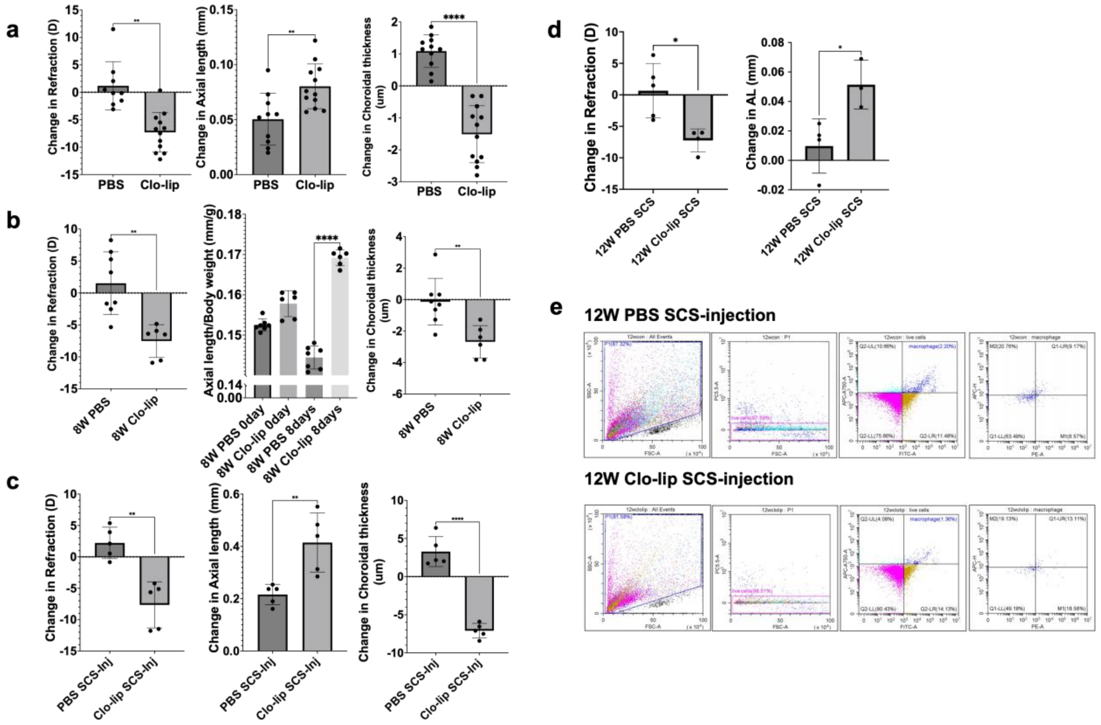

(a) Quantitative changes in Ref, AL, ChK in juvenile mice following systemic macrophage depletion (n=6 per group). Clo-lip treatment induced a significant myopic shift *(P < 0.01)*, axial elongation *(P < 0. 01)*, and choroidal thinning *(P < 0.0001)* compared with PBS controls. Data are presented as mean ± SEM. **(b)** Quantitative changes in Ref, AL, ChK in adult (8-week-old) mice following systemic macrophage depletion (n=4 per group). Clo-lip treatment resulted in a significant myopic shift *(P < 0.01)* and choroidal thinning *(P < 0.01)*, while axial elongation relative to body weight was increased *(P < 0.0001)* compared with PBS-treated mice. Data are shown as mean ± SEM. **(c)** Three-week-old wild-type C57BL/6 mice (n=5 per group) received SCS injections of PBS or Clo-lip following a defined dosing schedule that allowed corneal recovery and reagent absorption. Ref, AL, and ChK were assessed by SD-OCT. Clo-lip treatment resulted in a significant myopic shift *(P < 0.01),* axial length elongation *(P < 0.01)* and choroidal thinning *(P < 0.0001).* Data are shown as mean ± SEM. **(d)** Effects of SCS clodronate liposome injection on Ref and AL in adult (12-week-old) mice (n=5 per group). Compared with PBS SCS injection, Clo-lip SCS injection induced a significant myopic shift *(P < 0.05)* and axial elongation *(P < 0.05)*, Data are presented as mean ± SEM. **(e)** Flow cytometric analysis show macrophage populations in 12-week-old mice after PBS or clodronate liposome SCS injection, confirming effective macrophage depletion in the choroid. Data are presented as mean ± SEM. *P* values were determined using unpaired two-tailed t-tests/ *one-way ANOVA + Tukey post hoc*. **P* < 0.05. ***P* < 0.01. ****P* < 0.001. *****P* < 0.0001.

Supplementary Figure 2. Intraperitoneal LPS injection induces myopia-associated ocular changes and inflammatory and oxidative stress responses in the choroid

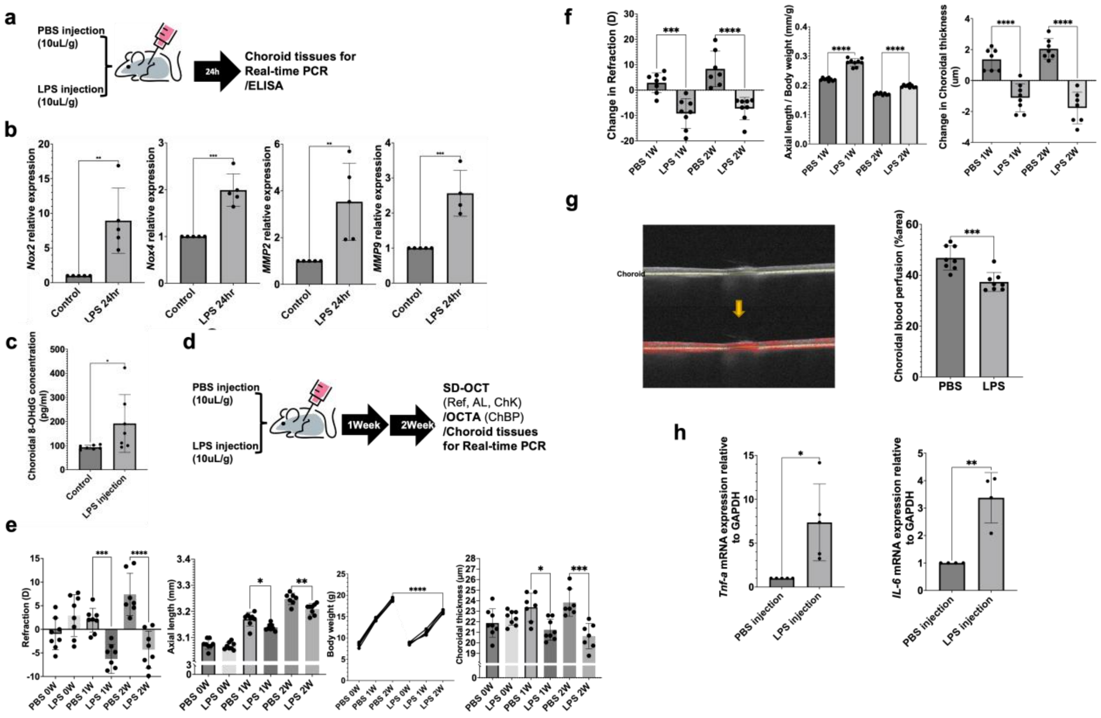

(a) Experimental design for acute inflammatory stimulation (n=5 per group). Mice received intraperitoneal injections of PBS or LPS, 10 μL/g, and choroidal tissues were collected 24 h later for real-time PCR and ELISA analysis. **(b)** Quantitative real-time PCR analysis shows significant upregulation of Nox2 *(P < 0. 01)*, Nox4 *(P < 0. 001)*, Mmp2 *(P < 0. 01)*, and Mmp9 *(P < 0. 001)* expression 24 h after LPS injection compared with PBS controls. Data are presented as mean ± SEM. **(c)** ELISA quantification of 8-OHdG. LPS injection significantly increased choroidal 8-OHdG concentration *(P < 0. 05)*, indicating enhanced oxidative DNA damage. **(d)** Experimental scheme for longitudinal assessment of LPS-induced ocular changes (n=8 per group). Mice received intraperitoneal PBS or LPS injections, followed by SD-OCT measurement of Ref, AL and ChK, OCTA assessment of choroidal blood perfusion (ChBP), and choroidal tissue collection for real-time PCR. **(e)** Repeated LPS administration induced a significant myopic shift *(P < 0. 0001)*, axial elongation *(P < 0. 01)*, and choroidal thinning *(P < 0. 001)*, Individual data points represent single eyes; bars indicate mean ± SEM. **(f)** LPS induced significantly greater changes in Ref *(P < 0. 0001)*, AL/BW *(P < 0. 0001)* and ChK *(P < 0. 0001)* compared with PBS controls at both 1 and 2 weeks. Data are presented as mean ± SEM.**(g)** Representative OCTA B-scan of the choroid with flow signal overlay. Structural OCT images are shown in grayscale, and blood flow signals are superimposed in red. The yellow dashed outline indicates the region used for choroidal blood perfusion analysis. LPS-treated mice exhibited a significant reduction in choroidal blood perfusion compared with PBS-treated controls *(P < 0. 001)*. **(h)** Real-time PCR analysis reveals significant upregulation of Tnf-α *(P < 0. 05)* and Il-6 *(P < 0. 01)* mRNA expression in LPS-treated mice compared with PBS controls. Data are presented as mean ± SEM. *P* values were determined using unpaired two-tailed t-tests/ *one-way ANOVA* + *Tukey post hoc*. **P* < 0.05. ***P* < 0.01. ****P* < 0.001. *****P* < 0.0001.

Supplementary Figure 3. SCS LPS injection induces oxidative stress in the choroid and impairs retinal function

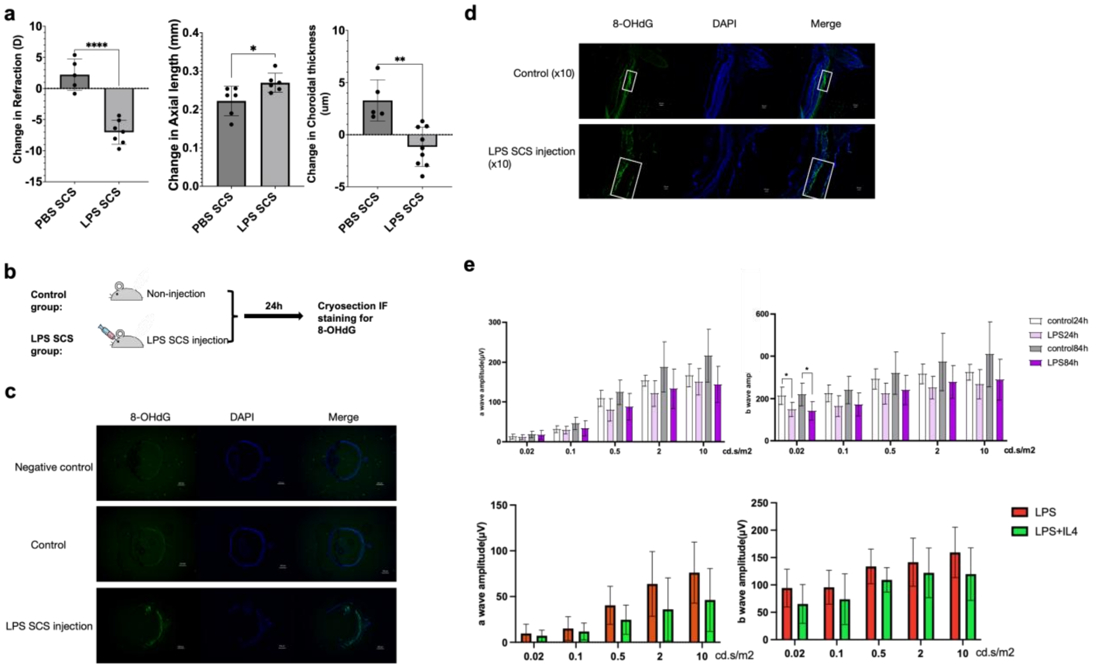

(a) Compared with PBS SCS injection, LPS SCS injection induced a significant myopic shift *(P < 0. 0001)*, axial elongation *(P < 0. 05)*, and choroidal thinning *(P < 0. 01)*. Data represent changes from baseline and are shown as mean ± SEM. **(b)** Experimental design for assessment of oxidative stress following LPS SCS injection. Eyes from non-injected control mice or mice receiving LPS SCS injection were collected 24 h later for cryosection immunofluorescence staining of 8-OHdG. **(c)** Representative immunofluorescence images of 8-OHdG staining in ocular cryosections. Minimal background signal was observed in negative controls. Compared with non-injected controls, LPS SCS injection markedly increased 8-OHdG immunoreactivity, indicating enhanced oxidative DNA damage. Scale bar:500μm. **(d)** Higher-magnification views (x10) of choroidal regions from control and LPS SCS-injected eyes. Enhanced 8-OHdG signal was predominantly localized to the choroidal layer following LPS SCS injection (boxed areas), further confirming choroid-enriched oxidative stress. Scale bar:50μm. **(e)** Electroretinography (ERG) analysis following LPS SCS injection. Scotopic a-wave and b-wave amplitudes were recorded at multiple light intensities. LPS SCS injection and treatment with IL-4 induced mild reductions in ERG responses. *P* values were determined using unpaired two-tailed t-tests. **P* < 0.05. ***P* < 0.01. ****P* < 0.001. *****P* < 0.0001.

Supplementary Figure 4. IL-4 administration promotes choroidal M2 polarization and STAT6 activation, accompanied by reduced oxidative stress

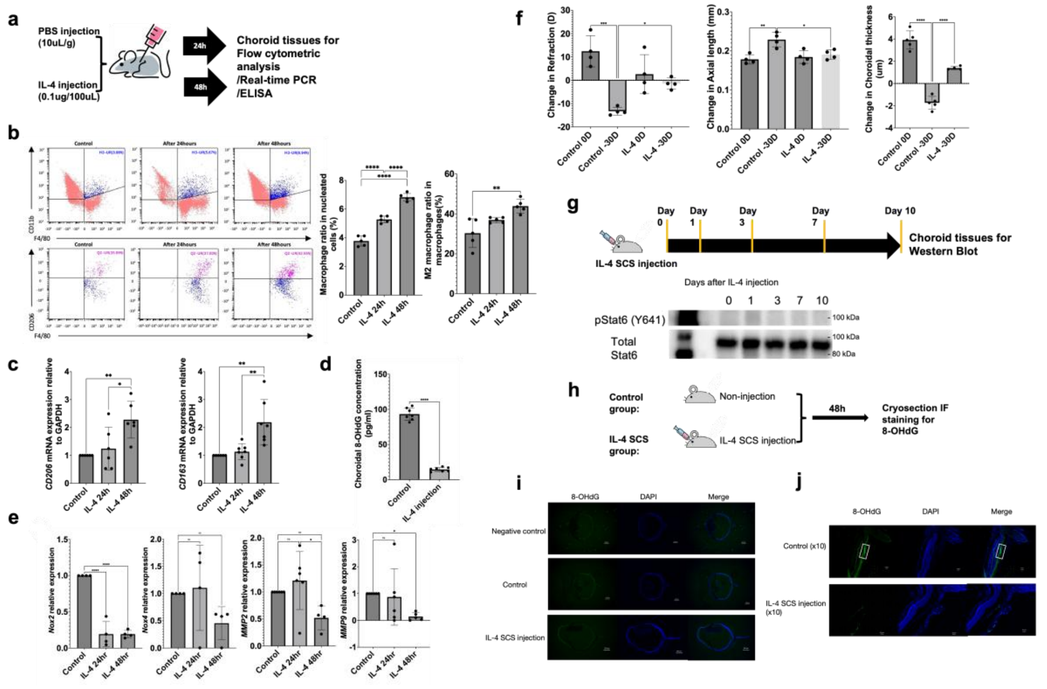

(a) Experimental design for acute IL-4 stimulation (n=5 per group). Mice received intraperitoneal injections of PBS or IL-4 (0.1 μg/100 μL). Choroidal tissues were collected 24 h and 48h after injection for flow cytometric analysis, real-time PCR and ELISA. **(b)** Flow cytometric analysis of choroidal macrophages following IL-4 treatment. Representative dot plots and quantification demonstrates a significant increase in the proportion of macrophages among nucleated choroidal cells *(P < 0. 0001)* and an elevated M2 macrophage ratio *(P < 0. 01)* following 48h of IL-4 treatment. **(c)** Real-time PCR analysis of M2-associated marker expression in the choroid. IL-4 treatment significantly upregulated Cd206 *(P < 0. 01)* and Cd163 *(P < 0. 01)* mRNA expression at 48 h compared with controls. Data are shown as mean ± SEM. **(d)** ELISA quantification of choroidal 8-hydroxy-2′-deoxyguanosine (8-OHdG). IL-4 injection significantly reduced 8-OHdG levels *(P < 0. 0001)*, indicating attenuation of oxidative DNA damage in the choroid. **(e)** Quantitative PCR analysis shows reduced expression of Nox2 *(P < 0. 0001)*, Nox4, Mmp2, and Mmp9 *(P < 0. 05)* following IL-4 administration. Data are shown as mean ± SEM. **(f)** Time-course analysis of STAT6 activation following SCS IL-4 injection. Western blot analysis of choroidal tissues demonstrates STAT6 phosphorylation in the choroid was markedly increased at 1 day after IL-4 SCS injection, while total STAT6 levels remain unchanged. **(g)** Experimental design for immunofluorescence assessment of oxidative stress following IL-4 SCS injection. Eyes from non-injected control mice and IL-4 SCS-treated mice were collected 48 h after injection for cryosection immunofluorescence staining. **(h)** Representative immunofluorescence images of 8-OHdG staining in ocular cryosections. **(i)** Compared with controls, IL-4 SCS injection markedly reduced 8-OHdG immunoreactivity. Scale bar:500μm. **(j)** Higher-magnification images of choroidal regions. IL-4 SCS injection significantly decreased 8-OHdG signal intensity in the choroid (boxed areas), confirming reduced oxidative stress at the tissue level. Scale bar:50μm. *P* values were determined using *one-way ANOVA + Tukey post hoc*. **P* < 0.05. ***P* < 0.01. ****P* < 0.001. *****P* < 0.0001.

Supplementary Figure 5. IL-13 administration promotes M2 macrophage polarization and improves myopia-associated choroidal alterations

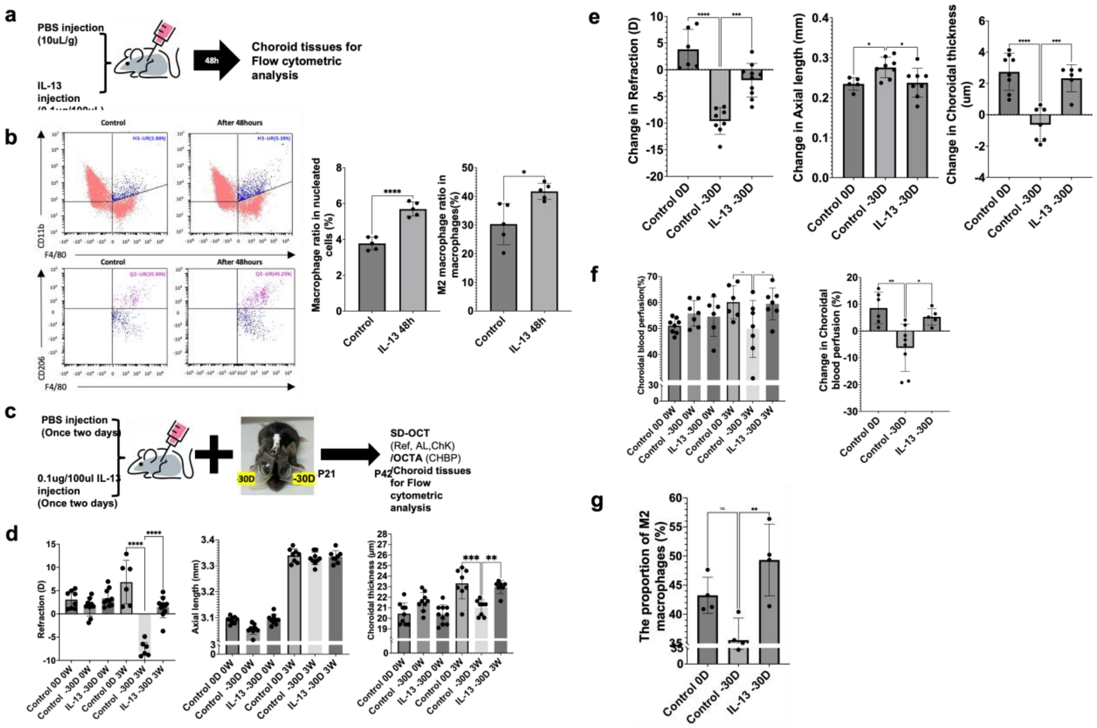

(a) Experimental design for acute IL-13 stimulation. Mice received intraperitoneal injections of PBS or IL-13 (0.1 μg/100 μL), and choroidal tissues were collected 48 h after injection for flow cytometric analysis. **(b)** Flow cytometric analysis of choroidal macrophages following IL-13 treatment. Quantification demonstrates an increased proportion of macrophages among nucleated choroidal cells *(P < 0. 0001)* and an elevated M2 macrophage ratio *(P < 0. 05)* compared with controls. **(c)** Experimental design for IL-13 intervention during LIM (n=5 per group). Mice undergoing LIM received intraperitoneal injections of PBS or IL-13 (0.1 μg/100 μL) once every two days from Day 21 to Day 42. Ocular biometry was assessed by SD-OCT, choroidal blood perfusion was measured by OCTA, and choroidal tissues were collected for flow cytometric analysis. **(d)** IL-13 administration significantly attenuated the LIM-induced myopic shift *(P < 0. 0001)* and partially preserved choroidal thickness *(P < 0. 01)*, Individual data points represent single eyes; bars indicate mean ± SEM. **(e)** Quantitative changes in ocular parameters during LIM. Compared with LIM-30D controls, IL-13 treatment reduced the magnitude of myopic shift *(P < 0. 001)*, axial length elongation *(P < 0. 05)* and choroidal thinning *(P < 0. 001).* **(f)** IL-13 treatment partially restored LIM-induced reduction in choroidal blood perfusion *(P < 0. 05)*. Right panel shows changes in choroidal blood perfusion relative to baseline. **(g)** Quantification of M2 macrophage proportion in the choroid during LIM. IL-13 treatment significantly increased the proportion of M2 macrophages *(P < 0. 01),* compared with LIM-30D controls. Data are shown as mean ± SEM. *P* values were determined using two-*way ANOVA* + *Tukey post hoc*. *$P < 0.05$. **$P < 0.01$. ***$P < 0.001$. ****$P < 0.0001$.

Supplementary Figure 6. Topical berberine administration attenuates lens-induced myopia progression in mice

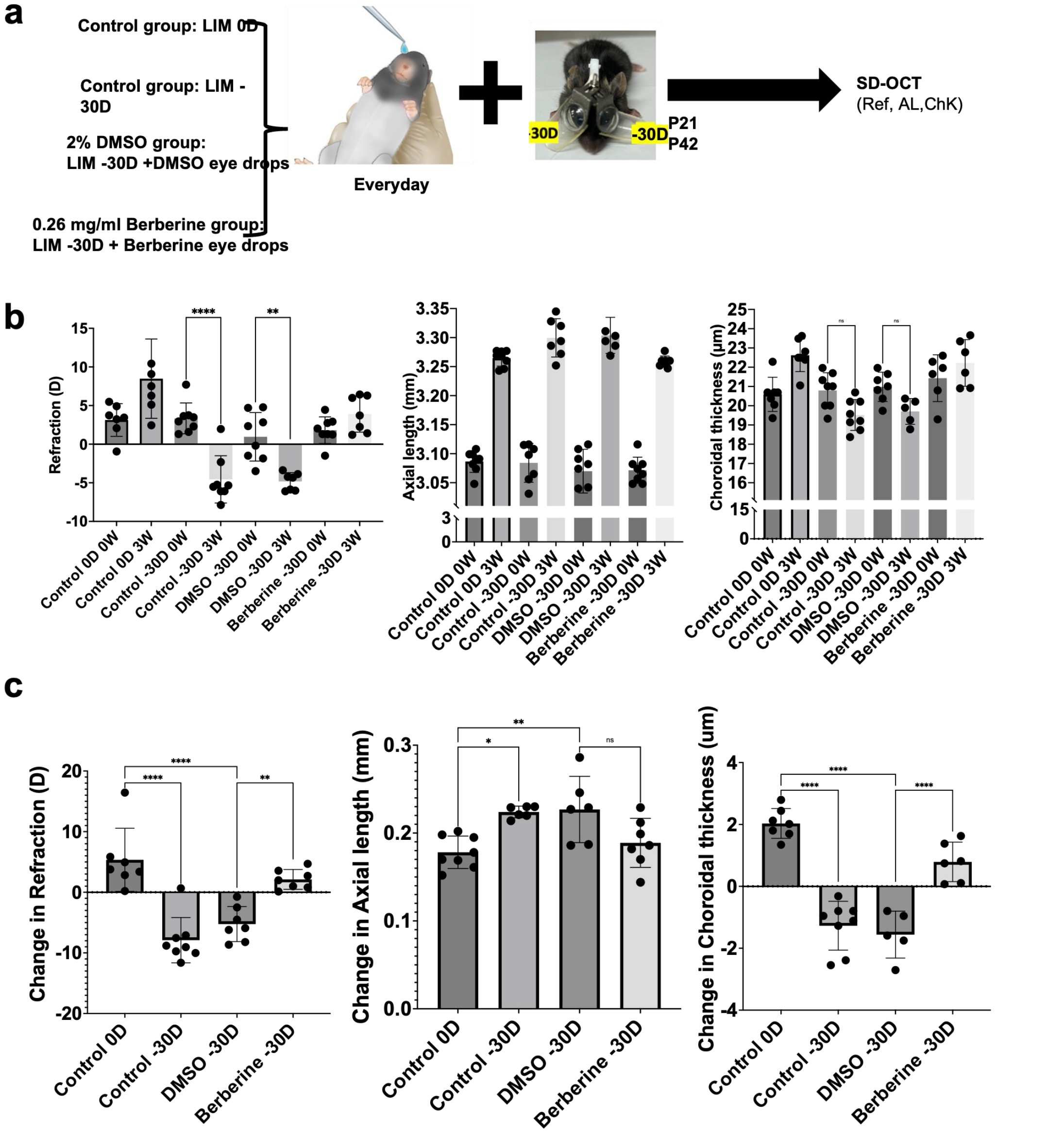

(a) Experimental design (n=4 per group). Mice were divided into four groups: control mice at baseline (LIM 0D), LIM -30D mice, LIM -30D mice treated with 2% DMSO eye drops, and LIM -30D mice treated with berberine eye drops (0.26 mg/mL). Eye drops were administered daily during the period of LIM. Ref, AL and ChK were assessed by SD-OCT. **(b)** Compared with untreated and DMSO-treated LIM mice, berberine-treated mice exhibited a significantly reduced myopic shift and axial elongation, while choroidal thickness showed no significant difference among treatment groups. Individual data points represent single eyes; bars indicate mean ± SEM. **(c)** Quantitative changes in ocular parameters relative to baseline. Berberine treatment significantly attenuated LIM-induced changes in refraction *(P < 0. 01)* and choroidal thinning *(P < 0. 0001)* compared with untreated and DMSO-treated LIM mice, whereas changes in axial length were modest. Data are presented as mean ± SEM. *P* values were determined using two-*way ANOVA* + *Tukey post hoc*. **P* < 0.05. ***P* < 0.01. ****P* < 0.001. *****P* < 0.0001.
